#  Human Lung Carcinoma Reaction against Metabolic Serum Deficiency Stress

**Published:** 2016

**Authors:** Maryam Nakhjavani, Nastaran Nikounezhad, Azadeh Ashtarinezhad, Farshad H. Shirazi

**Affiliations:** a *Department of Pharmaco/Toxicology, School of Pharmacy, Shahid Beheshti Medical University, Tehran, Iran.*; b *Pharmaceutical Sciences Research Center, Shahid Beheshti Medical University, Tehran, Iran.*; c *Occupational Health Research Center, School of Public Health, Iran University of Medical Sciences, Tehran, Iran.*; d *Department of Occupational Health, School of Public Health, Iran University of Medical Sciences, Tehran, Iran.*

**Keywords:** A549, Serum, Starvation, Cell Cycle, Metabolic, Stress

## Abstract

Cancer treatment is still of the greatest challenges that health care providers and patients are facing. One of the unsolved problems in cancer treatment is cells’ reaction to metabolic stress caused by harsh nutritional conditions around tumor. In order to be able to treat this disease properly, it is important to understand the true nature of the disease. In fact, the cells inside the central part of the tumor lack sufficient access to blood vessels, nutrients, and growth signals. After tumor shrinkage, the cells are exposed to favorable environmental conditions and might regrow and cause tumor recurrence. The main purpose of this study was to investigate the effect of serum starvation, as a type of metabolic stress, on human lung cancer cell line, A549. These cells were treated with 10% (control), 0.5% and 0.25% serum for 1 to 5 days. At 24 h intervals, the cells were released with 10% serum supplemented media. Starved or released cells were studied for their cycle and morphology. The results showed that the cells were actually arrested at G_1_ phase and following exposure to optimal conditions, the cells could be back to their cycle again. Furthermore, sub-G_1_ apoptotic cells population was not increased within the starvation period, while control cells had significant increase in sub-G_1_ cells. Morphological studies also showed that starved cells could make denser colonies while control cells were entering death phase. These observations provide some evidence for the generation of some effective resistance phenomena in cancer cells against harsh metabolic conditions.

## Introduction

Cancer is still one of the leading causes of death worldwide (1). Many investigators around the world are trying to look for newer therapeutics to cure the disease. However, prior to this step, it is better to get a deeper understanding about the biology of the tumor itself. 

Along with a tumor’s outgrowth, cancer cells are not distributed evenly in the tumor (2). The superficial tumor cells take the advantage of sufficient angiogenesis and enough access to space, nutrients, hormones, growth signals and oxygen, while central tumor cells not only bear more physical stress, but also lack adequate angiogenesis, and consequently insufficient nutrient and oxygen access (2). Following chemotherapy and tumor shrinkage these cells are once more exposed to optimal growth conditions, which may cause tumor re-growth. Tumor treatment with angiogenesis inhibitor agents also restricts tumor cells access to nutrients. 

There are many different protocols to treat cancer, including surgery, radiation therapy, chemotherapy, targeted therapy, laser and photodynamic therapy and etc (3). However, there are still high risks of cancer recurrence (4). As a tumor bears the treatment and shrinks, central tumor cells get sufficient access to favorable environmental conditions. Therefore, the tumor cells get the chance to be back to life and normal cycle once again and the tumorrecurs. 

The objective of this study was to investigate cancer cell cycle reaction to such metabolic stress. In order to induce metabolic stress, cells’ access to serum, as the source of amino acids and growth factor signals, became limited and cells’ response, in terms of morphology and cycle were studied.

## Material and methods

*Materials*

Human lung carcinoma (A549) (IBRCC10080) was provided by Iranian Biological Resource Center. Cell culture media (RPMI 1640), fetal bovine serum (FBS) and penicillin/streptomycin were purchased from Gibco BRL^®^. All other materials used in the study were purchased form Sigma^®^.

*Methods*

*Cell Culture and Treatment*

A549 cells were plated in culture dishes based on the methods previously described (5). Following reaching the plateau phase of growth curve, the supernatant media was discarded and the cells were completely washed with normal saline. The cells were then exposed to media containing 10% (control), 0.5% or 0.25% FBS for 1 to 5 days. On each day of the study, the cells were released in media containing 10% FBS. Later, starved or released cells were tested. 

*Cell Seeding Density Calculation*

In order to estimate the best cell seeding density, the cells were seeded in 4 different seeding densities (4000, 5000, 15000 and 50000 cells/cm^2^). The cell count was measured based on trypan blue assay, as described previously (6). 

*Morphological Studies*

In order to study the effect of starvation and release, cells’ morphology was studied at defined 24h intervals, using an inverted light microscope equipped with Moticam Pro camera, Motic^®^software.

*Cell Cycle Analysis*

To perform cell cycle analysis, starved or released cells were trypsinized and collected in cone tubes. 1 × 10^6^ cells per mL were centrifuged at 700 g, 4 °C for 10 min and washed twice with cold Ca^2+^/Mg^2+^ free Phosphate Buffered Saline (PBS). The cells were then fixed in ice-cold 70% ethanol and kept at -20 °C for at least 2 h. In order to stain the cells’ DNA, the staining solution (RNase A, triton X-100, Propidium Iodide) was dropped on the cell pellets and cells cycle was measured using flow cytometry (BD FACSCalibur^TM^, USA), and analyzed with FlowJo^®^ software, version 7.6.1.

*Statistics*

Cell cycle was analyzed using FlowJo^®^ software, version 7.6.1. Comparisons, when applicable, were measured by two-way analysis of variance, repeated measurement, using GraphPad Prism^®^, version 5.

## Results and Discussion

Cancer treatment is still an ongoing field of study for many of cancer researchers and one of the important issues in this area is reaching to better understanding of biology of the tumor cells to be equipped with more complete knowledge for improving more effective treatments. It has been widely believed that amino acids play a crucial role in cancer cell development (7). *In-vitro* cancer studies with cancer cell lines provide the cells with the required amino acid supply with adding fetal bovine serum, which also contains necessary growth signals for the cells to proliferate and survive. Serum restriction, on the other hand, has been a protocol for normal cell arrest in G_1_ phase of the cycle, a phenomenon called quiescence. G_1_-S transition has been known to need continuous stimulation by amino acids (8). In fact, Arthur B. Pardeecould for the first time explain the existence of restriction point(commonly known as R point) in G_1_, when the cells would check their environment for sufficient nutrients and growth signals to allow them to precede the cycle (9). Furthermore, high cell density and exposure to some chemicals have also been considered as contributing factors for quiescence induction (10, 11). However, cancer cells’ behavior is thought to be different from normal ones. Within normal to cancer cell transformation, various capabilities are acquired including limitless proliferation potential, avoiding apoptosis and resisting death, metastasis, insensitivity to anti-growth signals and etc (12, 13). Therefore, in suboptimal environmental conditions, cancer cells may have different behavior profiles compared to normal cells. 

**Figure 1:**
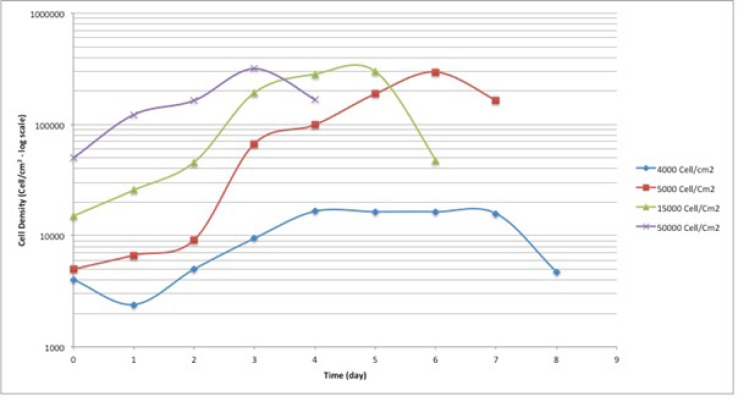
Cell seeding density calculation for A549 cell line. The cells were seeded at 4 different seeding densities; 4000, 5000, 15000 and 20000 cell/cm2.

**Figure 2.**
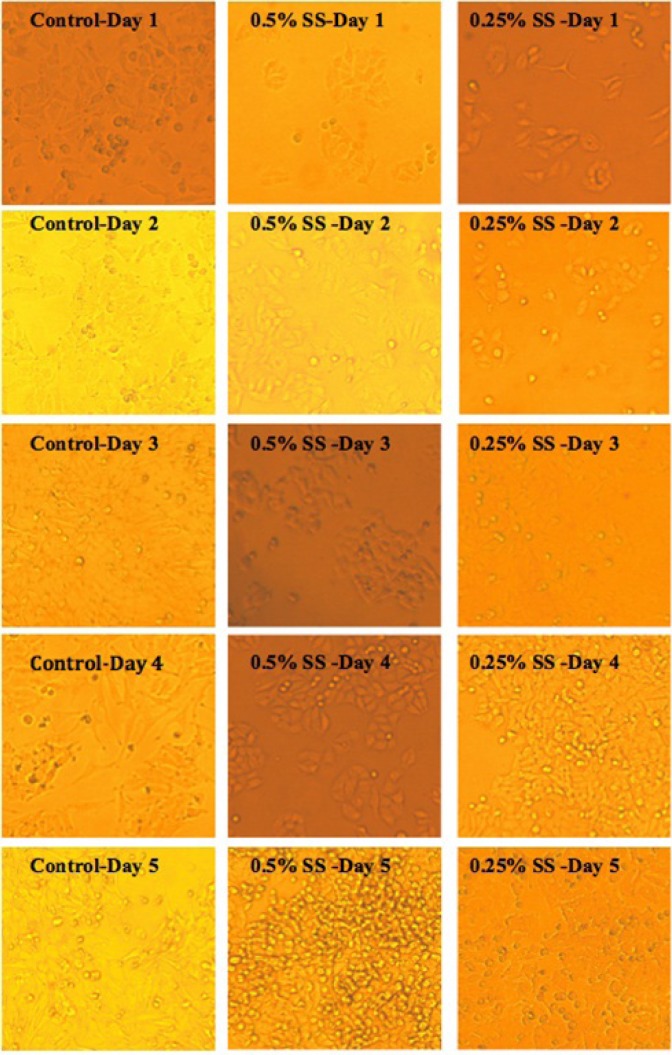
Morphological study on A549 cell line, following exposure to 10% serum (control), and starved stated at 0.5% and 0.25% serum. In the images below, SS indicates “Serum Starvation”.

**Figure 3.**
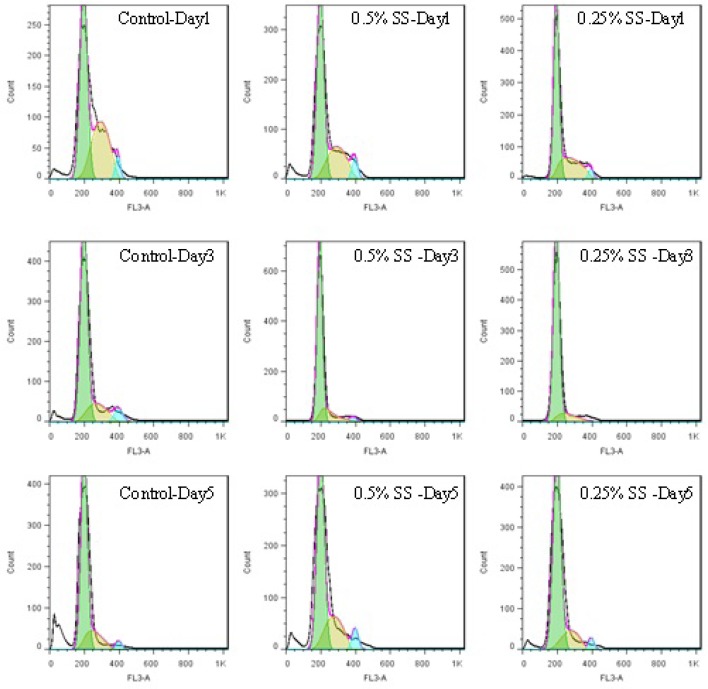
Histograms of cell cycle of A549 cell line, in a 5-day study. The cells were starved for 1 to 5 days in media containing 0.25% and 0.5% serum, while 10% serum was considered as control. In each day of the study, the cycle of the cells was analyzed using flow cytometry

**Figure 4:**
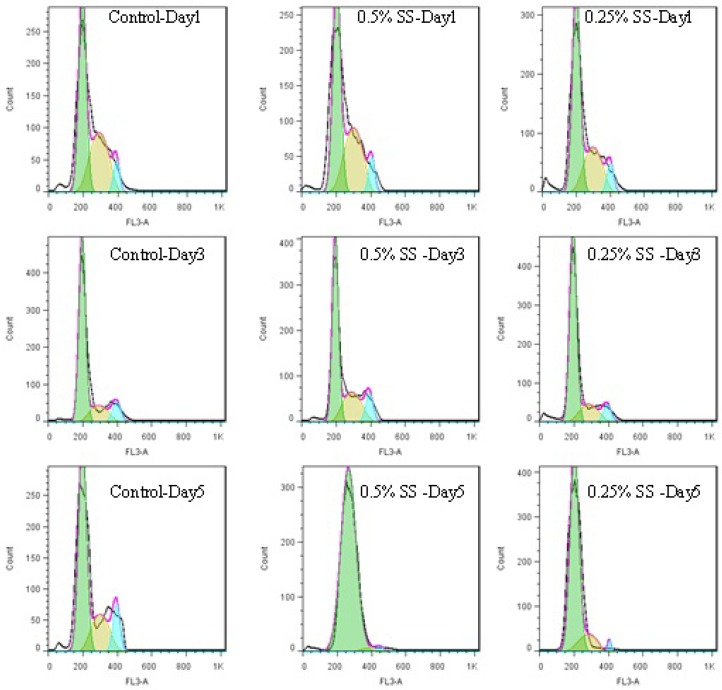
Histograms of cell cycle of A549 cell line, released after starvation in a 5-day study. The cells were starved for 1 to 5 days in media containing 0.25% and 0.5% serum, while 10% serum was considered as control. In each day of the study, the cell were released in media containing 10% serum

**Figure 5:**
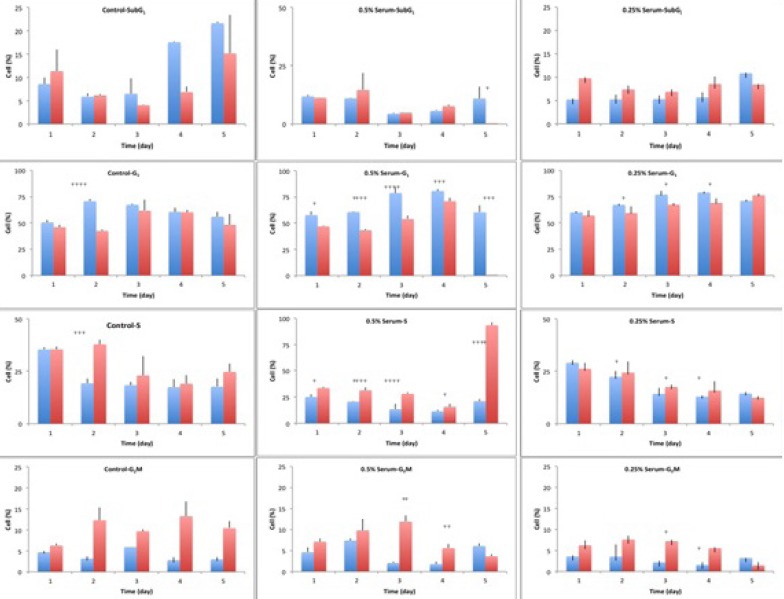
Cell cycle analysis of A549 cells, in a 5-day study, at starvation and release status. The cells were starved for 1 to 6 days in media containing 0%, 0.25% and 0.5% serum, while 10% serum was considered as control. In each day of the study, the cells were released in media containing 10% serum, for 24 hours. The cycle of the cells was analyzed using flow cytometry. In each graph, blue columns indicate starvation and the red columns show the release status of the respective cell, after 24 hours of release.The significance level was considered as *p*<0.05

In the current study, the cell cycle changes of human lung adenocarcinoma cell line, A549, to harsh environmental conditions; in terms of serum restriction was investigated at 0.5% and 0.25% serum. Based on the results of cell seeding density calculations, the cells were seeded at the density of 4000 cells/cm^2^. At this seeding density, the cells had the longest plateau phase, which was suitable for long-term study (Figure 1.). Therefore, the starting point of the experiment was the 4^th^ day after seeding the cells. The cells were at plateau phase to mimic the cells’ state deep inside the central parts of a tumor, in terms of cell density.

Control cells with sufficient access to optimal environmental conditions go on their natural cycle. The histograms of cells cycle are illustrated in Figure 3. Based on the results illustrated in **Table 1**. sub-G_1_ control cells populationdo not make statistically significant changes in the first 3 days of the study, while the sub-G_1_populationincreaseson the last 2 days of the study, as the cells enter their death phase. Control G_1_ cells’ population is increased by day 2 of the study (*p*<0.0001) while the S phase cells population decreases at the same time. From day 3 onwards, as the cells start to slowly die, the G_1_ population starts gradual decreasing and sub-G_1_ increases. Knowing that the G_2_/M cells’ population also do not change statistically, it is possible to conclude that the cells are arrested at G_1_-S transition on day 2 of the study. The possible explanation for this observation might be confluency, asconfluency had been shown to play a role in G_1_ accumulation in normal cells (14). It is possible that some similar mechanisms play role in the observed G_1_ accumulation in this cancer cell line. It can be explained that the cells accumulate in G_1_, however, after day 2 of the study, G_1_ arrested cells enter the death phase and sub-G_1_ apoptotic status. Confluent A549 colonies are observable in Figure 2.

 For the test cells grown in 0.5% and 0.25% FBS, this arrest happens on day 3 (*p*<0.0001). At 0.5% serum starvation, G_1_ accumulation lasts for 2 days, while at 0.25% serum starvation, no statistically significant changes in cell population of different phases is detected. It seems that the harsher the conditions become, the more resistant the cells react. The other evidence to this statement is the lack of sub-G_1_ increase in starved status cells. Furthermore, the morphological studies show that along with the starvation period, by the time control cells pass the final days of their growth curve, starved cells are making denser colonies. In fact this observation is in accordance with other evidences that state fasting imposes further tumor cell proliferation(15).

Exchanging the cells media with media completed with 10% FBS for 24 h, could cause decreasing the cells population in G_1_ phase and increasing the cells’ population in S and G_2_M phases ( 4. and 5.). In other words, the cells were released from the arrest. The general pattern of release in starved cells shows growth factor dependency of the cells to go on their cycle. Therefore, it is possible that some mechanisms similar to R-point (8) in normal cells exist in cancer cells too, which sensitize them to the environmental conditions. Releasing A549 cells with media containing 10% serum showed that the damage to the remaining cells was not irreversible with regard to the cell cycle, but the cells could still go on through their cycle. 

This shift in cells accumulation happened only on day 2 for the control cells. Test cells grown in 0.5% FBS showed better release pattern compared to cells grown in 0.25% serum. The possible reason for this might be explained by Lum’s research. He believed that stress induced on the cells within the starvation period leaves the cells requiring longer time for recovery and for re-synthesis of intracellular organelles (16). As 0.25% starved cells underwent harsher environmental conditions, it is possible that they require longer time for a better release. 

One of the interesting observations in this study was that despite the metabolic stress induced on the cells and the observed G_1_ accumulation, no statistically significant change happened in the sub-G_1_ population, as a marker of apoptotic cells. There are many compounds that induce G_1_/G_0_ arrest in A549 cell line and apoptosis arises along with the arrest, even in short term exposure to the compounds as long as 24 to 72 h (18). The levels of various intracellular molecules change based on the exposed compound, including decrease in levels of cdc2 and cdc4, cdk2, cdk4, cdk6 and cyclin-D1 and cyclin-D3, increase in the level of p27^KIP1^, Rb2/p130 and also the activation of apoptosis inducing caspases and biomolecules (18). Their observations approve the G_1_ arrest and the observed increased sub-G_1_ population. However, the results of our study basically lack such increase in sub-G_1_populationin starved cells. 

In general, there are some imaginable explanations for the observed effects; autophagy is one of the probable subjects that needs to be studied in such starved cells. Since amino acid starvation is known to trigger autophagy (17), a pathway that the cells use to degrade their intracellular organelles as a source of energy for longer survival (18). Therefore such cells would not go through apoptosis, unless autophagy pathways are defected. 

In conclusion, to address one of the main questions about the survival and recurrence of cancer tumor cells even after intensive chemotherapy programs that include anti-angiogenesis agents, we have studied the adaptability of the human lung adenocaarcinoma cell line, A549, to harsh environmental conditions in this study. We assessed the impact of starvation on this cancer cell line. As described in the results and discussion sections, starvation does not seem to have a very dramatic effect on the cellular survival, as the cells react in a very complex way. We have shown good cell survival and cell functional abilities even after 5 days of starvation. Our results have confirmed recurrence of cellular cycling after the release of cells from starvation-imposed harsh condition. This may help explain aggressive re-growth of cancers after initial response to chemotherapy combined with anti-angiogenic agents, which restrict cells’ access to sufficient nutrients.
